# Probabilistic Analysis of a French Legionellosis Outbreak Shows Potential Role of Wastewater Basin

**DOI:** 10.3390/microorganisms10020422

**Published:** 2022-02-11

**Authors:** France Wallet, Leila Fontenay, Pierre-André Cabanes

**Affiliations:** 1 EDF-Services des Etudes Médicales, 4 Rue Floréal, 75017 Paris, France; pierre-andre.cabanes@edf.fr; 2 Société de Calcul Mathématique, 111 Faubourg Saint Honoré, 75008 Paris, France; leila.fontenay@gmail.com

**Keywords:** air-borne infections, epidemiology, *Legionella*, legionellosis, Legionnaires’ disease, outbreaks

## Abstract

Based on the data from a French outbreak of legionellosis, a probabilistic approach was developed to analyze and assess the potential role of several suspected sources of contamination. Potential dates of exposure of all cases were determined using back-calculation, using two probability distribution functions to model incubation period. A probabilistic analysis and risk assessment were then used to determine the most probable sources of contamination for each wave of the outbreak. The risk assessment was based on parameters representing emission and dispersion of *Legionella*: level and duration of emission; aerosol dispersion capacity; and probability of potential exposure for each patient. Four types of facilities containing the *Legionella* epidemic strain were analyzed: cooling towers, aerated wastewater basins, high pressure water cleaners, and car wash stations. The results highlighted the potential role of an aerated wastewater basin in the outbreak in addition to cooling towers. The role of high-pressure water cleaners and car wash stations appeared to be non-significant. This study also reveals the lack of knowledge on facility parameters that can be useful for microbial risk assessments. This type of probabilistic analysis can be used to quantitatively assess the risk for various facilities in order to manage a legionellosis outbreak.

## 1. Introduction

Some species of bacteria belonging to the *Legionella* genus, especially *Legionella pneumophila* serotype 1 (Lp-1), are recognized as a cause of community-acquired and nosocomial pneumonia known as Legionnaires’ disease or legionellosis by inhalation of contaminated water aerosols [1]. Community-acquired legionellosis is associated with a high case-fatality rate of around 6–11% [2,3,4,5]; however, case-fatality rates of over 30% have been reported for nosocomial legionellosis [3], notably in patients with specific risk factors [6,7]. Surveillance studies indicate that legionellosis is an increasing public health concern in many countries worldwide [8,9].

The bacteria causing legionellosis grow in natural or artificial aquatic environments with the highest levels of contamination at water temperatures between 30 and 50 °C [10]. These bacteria are also able to invade and survive within various species of protozoa [11]. The most common sources of human contamination are facilities that produce fine water droplets or aerosols <5 μm in size containing the bacteria, including shower heads, cooling towers, mist generators, spa pools/thermal baths, and decorative fountains [1,12]. These fine aerosols are inhaled into the pulmonary alveoli and induce infection [13]. Public health measures for the prevention of legionellosis are based on the concept of risk assessment through close environmental surveillance and monitoring, combined with low-emission cleaning and disinfection procedures [5,12,14,15].

The aim of this study was to analyze and assess the potential sources of the outbreak, taking into account factors linked to the operation of these facilities [16]. A new methodology using a probabilistic approach was applied to a well-documented outbreak of legionellosis that occurred in France in 2003–2004 [17,18,19].

## 2. Material and Methods

### 2.1. Source of Data: Legionellosis Outbreak

The data used for this probabilistic analysis came from the largest outbreak of legionellosis to have occurred in France to date. The characteristics of the patients involved in this outbreak have been described in detail previously [19]. Briefly, 86 patients in the Pas-de-Calais region of France were diagnosed with legionellosis between 5 November 2003 and 22 January 2004. Eighty-four cases were diagnosed by urinary antigen and two cases by seroconversion. Over half, 60% (52/86), were male and the median age was 75.5 years. Of the 86 cases, 18 patients died (21%). Nearly all patients, 97% (83/86), lived within a 12 km radius of Harnes, corresponding to an incidence of 3.9 cases/10,000 inhabitants. The three remaining cases had travelled to the same area. Positive culture was found for 23 of the 86 cases, with a common strain *L. pneumophila* serotype 1 named the Lens strain (Lp-1 Lens), the epidemic strain.

An environmental investigation carried out by the Regional Office of the French Ministry of Industry, Research and the Environment helped list and inspect all potential sources of contamination for identifying the source of the outbreak [18]. A microbiological investigation conducted by the French National Centre for *Legionella* analyzed clinical and environmental samples, including atmospheric samples, from all potential contamination sites. All isolated strains were identified using pulsed field gel electrophoresis (PFGE). The epidemic *L. pneumophila* serotype 1 Lens strain (Lp-1 Lens) was found in four types of facilities: two factory cooling towers, an aerated wastewater/sludge basin, a car wash station, and a high-pressure water cleaner used on cooling towers that may have aerosolized *Legionella*-contaminated biofilms [19]. Most of these facilities were located on the same industrial site: the two cooling towers, the wastewater/sludge basin, and the high-pressure water cleaner, the latter using chlorinated water for cleaning some elements of the cooling towers. The car washing station was located near the industrial site in the same town. These four facilities could be potential contamination sources in all or some of the cases. After investigation, the cooling towers were officially declared as the source of the contamination, even if they were not in operation throughout the outbreak. All epidemiological data concerning the outbreak were collected from the Departmental Office of Health and Social Affairs and Regional Office of Epidemiology.

### 2.2. Back-Calculation of Date of Exposure

The first step of the method consisted of the determination of the probability distribution of the potential date of exposure for all cases, based on knowledge of the legionellosis incubation period, which corresponded to 2–10 days between exposure to the bacteria and onset of the first symptoms of the disease.

We used back-calculation, as previously developed, with incubation period characterized by a probability distribution function (PDF) [20]. After testing numerous PDFs, we retained a uniform distribution between 2 and 10 and a lognormal distribution function with a mean of 2 days and a standard deviation of 2.74. The parameters selected are compatible with the known virulence of the *Legionella* strain involved in this outbreak.

The probability distribution of the date of potential exposure was then divided into three waves determined for each PDF used, and uniform and lognormal distribution functions.

### 2.3. Probability of Exposure to the Potential Sources of Contamination

Using information on the facilities from this outbreak, we collected the periods of operation (days and hours) corresponding to aerosol emissions for the four potential sources of contamination where the epidemic strain was found.

Combined with the potential date of exposure of each patient, we determined the number of patients potentially exposed to each source and assessed the probability of exposure to the various potential sources of contamination.

### 2.4. Risk Assessment of Each Source of Contamination

To determine the most likely source of the outbreak and categorize the facilities according to their potential role in the epidemic, we calculated a relative risk for each source of contamination based on parameters representing emission and dispersion of *Legionella*: the level and duration of emission of *Legionella* in the atmosphere using characteristics of each facility (contamination, operating characteristics); the aerosol dispersion capacity, which depends on the speed at which aerosol is emitted (low for the wastewater aerator and car wash, moderate for the high pressure cleaner, and high for the cooling-tower); and probability of potential exposure for each patient. For cooling towers, data on flow characteristics were taken from technical reports on the epidemic [21]. The wastewater basins were equipped with several fast turbine surface aerators, with water being thrown into the air by the action of the turbine. Based on data from the technical report [18], we assessed the flow rate by inverse dispersion modeling using a single-source Gaussian plume model (Screen 3 software, US-EPA) based on the air concentration measured with a liquid cyclonic impactor on 14 January 2004 at 0 and 200 m downwind of the basin, at 5400 and 330 CFU/m^3^, respectively [18]. Table 1 summarizes the data used and assumptions made to perform the calculation of the flow of *Legionella* into the atmosphere for the various potential sources.

## 3. Results

### 3.1. Outbreak in Three Waves

**Figure 1:**
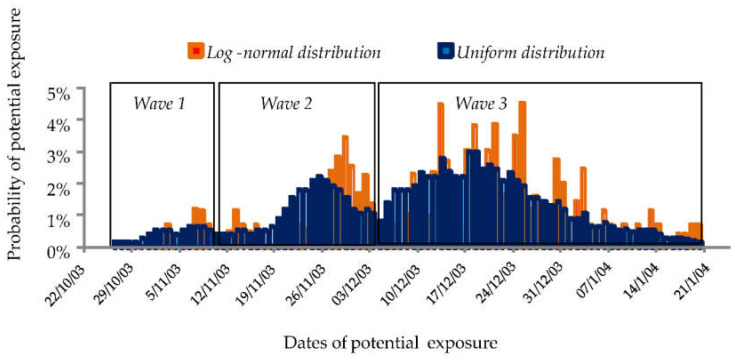
Distribution of potential dates of exposure using two laws of probability: truncated lognormal distribution and uniform distribution.

Distribution of the potential day of exposure for the 86 patients after consideration of the incubation period revealed three separate curves corresponding to three successive waves of infection during the outbreak, and not two as indicated in the original report [19]. Figure 1 shows the results produced using two probability distribution functions to model the incubation period: a uniform distribution and a truncated lognormal distribution for an incubation period of 2 days at least and 10 days at most.

### 3.2. Probability of Exposure to the Potential Sources of Contamination

In this part of the investigation, we examined all four facilities containing water contaminated with Lp-1 Lens and dispersing aerosols, namely cooling towers, a wastewater basin, a car wash station, and a high-pressure water cleaner. The analysis revealed that, for the three waves together, 35% (30/86) of all cases (uniform law) and 42% (36/86) of all cases (lognormal law) had a zero probability of having been exposed to the cooling tower aerosols (Table 2). In contrast, 100% of all 86 cases (uniform law and lognormal law) had a nonzero probability of having been exposed to aerosols released by the wastewater basin.

### 3.3. Risk Assessment of Each Source of Contamination

#### 3.3.1. First Wave of the Outbreak

A combination of two potential sources (the cooling tower and wastewater basin) was the most likely source of contamination during this first wave of infection (Table 3).

#### 3.3.2. Second Wave of the Outbreak

The cooling tower was the most likely source of contamination during this second wave (Table 3). However, for the uniform distribution, investigation of the cooling tower shows that 12% (3/26) of the 26 cases in this wave had a zero probability of having been exposed and were likely to have been infected by the cooling tower (Table 2). In other words, in this case, the cooling tower could not be the only source of infection in the second wave of the outbreak.

#### 3.3.3. Third Wave of the Outbreak

In this wave, the wastewater basin was the most likely (97%) source of contamination (Table 3). Investigation of this source demonstrated that 100% of the cases (53 cases for the uniform distribution and 59 cases for the lognormal distribution) in this wave had a nonzero probability of having been exposed and were likely to have been infected by this source (Table 2).

## 4. Discussion

Despite in-depth investigations, numerous legionellosis outbreaks are unresolved, showing that determining the origin of an outbreak is very difficult. Therefore, it is very important to determine the exposure period, and thus to estimate the source of the epidemic with more accuracy.

This study aimed to determine the most likely sources of legionellosis outbreak and was applied to a well-documented outbreak where several potential sources were suspected.

The first step of this methodology is based on a back-calculation of the date of exposure. We chose an incubation period of 2 to 10 days modeled by two different probability distributions: uniform and log-normal density functions. The incubation period taken into account seems realistic because 90% of the incubation periods would be within this range [22]. Longer incubation periods have been described for legionellosis; however, the virulence of the strain involved in the outbreak studied [23] is compatible with the selected incubation period. Egan used a uniform PDF [20], and log-normal PDFs were historically used, to model incubation periods [24,25,26]. Few works are available on PDFs that can model the incubation period of legionellosis and the associated parameters [22]. The back-calculation method used does not require using the hypothesis of the distribution of *Legionella* release, because the hypothesis is recognized as being too simplistic [22].

The uncertainty associated with this first step of our methodology concerns the duration and the probability distribution of the incubation period that could be linked to the virulence of the epidemic strain. Comparative *Legionella* genome analysis should provide answers [27]. Knowledge of the exact date of onset of the clinical signs is important for the accuracy of this step.

Retained risk facilities are those where the environmental investigation found the presence of the epidemic strain Lp-1 Lens and a concentration of *Legionella* higher than the legal target threshold if it exists (10^3^ CFU/L for cooling towers). All potentially involved facilities may not have been sampled; however, the scale of the epidemic resulted in an unprecedented environmental study and many potential sources were investigated. This means we can be relatively confident that the environmental investigation was comprehensive.

Knowledge of the operating times of the various facilities potentially involved has enabled us to determine the cases that could not be exposed to any of the facilities identified as potentially causing contamination. Using probabilistic modeling, our current study shows that the cooling towers could not have been the only source of infection, and that the aerated wastewater basin, which also contained the infection strain Lp-1 Lens, may have provided uninterrupted *Legionella* contamination and may have been responsible for most of the cases in the third wave. Indeed, the original study concluded that the outbreak began to die out after final cooling tower closure, but it was over only after the wastewater basin ventilators had been disconnected [19]. Based on the operating characteristics of the facilities, the cooling towers and high-pressure water cleaners alone cannot explain all the cases in the outbreak. The wastewater basin is the only facility likely to explain all the outbreak cases. Surprisingly, this facility was not seen at the time as possibly causing cases, despite the measurement of a high concentration of *Legionella* downwind of the wastewater basin, while the cooling towers were stopped. This may be due to the fact that no cases of legionellosis were described at the time as being related to this type of facility. The direct involvement of aerated wastewater treatment ponds in outbreaks of Legionnaires’ disease was reported after this period [28,29,30]. Kusnetsov et al. reported a case of Legionnaires’ disease associated with an industrial wastewater treatment system used by the forestry industry in Finland [29], and Olsen et al. described wastewater aeration ponds as the main source of contamination during three outbreaks of legionellosis in Norway in 2005 and 2008 [30]. These studies and our current study emphasize the need to recognize this potential source of *Legionella* infection, and to take action to control *Legionella* concentrations and to prevent the release of wastewater containing *Legionella* into the environment [30]. Contamination by *Legionella* of this kind of facility and the presence of *Legionella* in the air, and therefore its possible dissemination, has been described [30,31,32,33,34,35,36].

The original epidemiological study of the French legionellosis outbreak concluded that the two waves of infection probably corresponded to an initial continuous plume of *Legionella* dispersion from the cooling tower, and then a second infection wave corresponded to aerosol emission resulting from high-pressure cleaning and restarting of the cooling tower.

Our study suggests that the outbreak took place in three successive phases (waves), unlike the report published during the investigation into the epidemic (two waves). Our analysis therefore focused on the three phases that may correspond to three different source facilities. Our study attempted to go further by prioritizing the facilities potentially responsible for Legionnaires’ disease cases according to parameters of emission and dispersion of *Legionella*.

For the wastewater basin, we used an inverse model based on air concentration measurements. Even if measurement of *Legionella* in air is not yet scientifically approved, the concentrations measured show a *Legionella* air concentration never before seen elsewhere. Some studies have measured or modeled the quantity of aerosols emitted by these facilities [36,37,38] and could explain the high concentrations of *Legionella* measured in the air due to the high concentration of *Legionella* found in the wastewater basin.

Regarding the car washing facility, we assumed that all water flows were aerosolized, which is an unrealistic and much exaggerated assumption, made in the absence of data. Some experimental data showed that aerosolization was actually much lower [39,40]. A study of spray exposure could provide information on the quantity of aerosols inhaled [41]. Even with these much-exaggerated assumptions, the results of our study show that the role of this facility appears to be insignificant.

For the high-pressure water cleaner, no *Legionella* were found in the water used; however, the assumption here is that biofilms containing *Legionella* released particles during cleaning of the cooling tower elements. Due to the lack of data on this phenomenon, we made some subjective assumptions. The data on cooling towers are based on the data collected during the epidemic by experts [21]. Experiments can be used to refine this data [42]. The parameter used to describe the passage of *Legionella* into the atmosphere tends to attribute a greater role to facilities that emit a large quantity of aerosols due to the coefficients used. Therefore, this is the main parameter.

The aerosol dispersion capacity parameter is highly arbitrary but takes into account the speed and the height of the facilities, parameters that can influence the dispersion of *Legionella*. The levels used tend to minimize the role of the wastewater basin, but were in accordance with the limited published data, which are not consistent with on-site measurements [31]. Lack of data on wastewater basin contamination at the beginning of the outbreak may explain the low relative risk of this facility in the second wave of the outbreak. Other parameters can be integrated, such as the possibility of aggregates of *Legionella*, but no data are available on this parameter for any type of facility. The coefficient used for cooling towers may have contributed to their greater role in wave 2 of the epidemic. The role of high pressure cleaners appears insignificant despite the high dispersion capacity used, in keeping with the published data [39,40,43]. The other parameters (duration of aerosol emission, potentially exposed patient) take into account the objective data of the operating characteristics of the facilities concerned and the exposure data according to the date of exposure.

In this work, we considered only direct *Legionella* transmission from the selected potential sources. Possible secondary dissemination of the epidemic *Legionella* strain by contamination of another facility, such as a cooling tower [44], which would then contaminate people and could explain the cases very far from the source of exposure [19], was not taken into consideration. We also did not consider the risk factors (age, silicosis, etc.). This could be a future improvement to this method.

This study could also be improved by taking into account the aggregate phenomenon, a more or less realistic possibility according to the facilities to disperse aggregates of *Legionella* in aerosols rather than *Legionella* alone [45]. We did not take into account the survival of *Legionella* in the air during aerosol dispersion, which may be modified by the elements present in the aqueous medium, in particular for the wastewater basin [46].

The second step is based on the accuracy of the data on high-risk facilities. Monitoring of these facilities, which is the basis of the framework in many countries [16], is expected to recover such important data.

Despite numerous uncertainties, this method can be useful in determining which facilities are most likely to cause epidemics, with sufficient information on the operating characteristics of the facilities. Studies are required to generate data on the aerosolization capabilities of the various hazardous facilities.

Our study highlighted the potentially important role of aerated wastewater basin in the outbreak. Microbial risk assessment is a useful tool for further investigating the potential role of such facilities in *Legionella* contamination [47]. However, *Legionella* risk assessment is fraught with many uncertainties, including strain infectivity, infectious dose, parameters of aerosolization for each facility, and survival of the bacteria in the air, which is correlated to water quality [47,48].

## 5. Conclusions

Probabilistic analysis of data from a legionellosis outbreak is a possible means of assessing the potential source that contributed to an outbreak. This analysis requires a prior full environmental investigation, identifying all possible sources of infection (potentially contaminated facilities emitting aerosols), their level of contamination, and their history of operation. At this time, however, the study is limited by the fact that many data related to the operation of facilities, such as car washes, high-pressure water cleaners, and wastewater treatment systems (*Legionella* flow, dispersion), are not sufficiently accurate. We also did not take into consideration the possibility that *Legionella* may have been present in biofilms, a parameter that may be affected according to the type of facility and that could play a role in *Legionella* survival and transmission [31,49,50]. Nevertheless, this type of analysis can help us to learn more about these phenomena, and can be used to quantitatively assess risk for a given facility so that appropriate control methods can be implemented [47,51]. The probabilistic analysis of potential sources of infection may produce important and robust results; this method could be usefully implemented for other outbreaks.

## Figures and Tables

**Table 1:** Level of contamination with *Legionella* and emission into the atmosphere for each potential source of infection.

Facility	Characteristics (L/h)	Contamination (CFU/L)	*Legionella* Emission (CFU/s)	Assumptions
Car wash	500	1600	200	The entire flow was aerosolized
High-pressure water cleaner	500	100,000	700	5% of the flow aerosolized biofilm
Cooling tower	90	100,000	5000	Aerosol emission calculated for the two cooling towers
Wastewater treatment plant	1.8	200,000,000	100,000	Inverse dispersion modeling using single source Gaussian plume model based on air concentrations measurement

**Table 2:** Probability of exposure to the different potential sources of contamination for each wave of the outbreak.

Probability Distribution Function	Wave of Outbreak	Duration (Days)	Start Date–End Date of the Wave	Number of Cases	Cases with a Nonzero Probability of Exposure to the Source (Number of Cases—% of Cases Per Wave)			
					Cooling Tower	High-Pressure Water Cleaner	Wastewater Basin	Car Wash Station
Uniform distribution	Wave 1	16	26 October 2003 to 10 November 2003	7	7 (100%)		7 (100%)	
	Wave 2	23	11 November 2003 to 3 December 2003	26	23 (88%)		26 (100%)	
	Wave 3	50	4 December 2003 to 22 January 2004	53	26 (49%)	32 (60%)	53 (100%)	33 (62%)
	Total duration of outbreak	89	26 October 2003 to 22 January 2004	86	56 (65%)	32 (37%)	86 (100%)	33 (38%)
Lognormal distribution	Wave 1	9	1 November 2003 to 9 November 2003	4	4 (100%)		4 (100%)	
	Wave 2	23	11 November 2003 to 3 December 2003	23	23 (100%)		23 (100%)	
	Wave 3	50	4 December 2003 to 22 January 2004	59	23 (39%)	16 (27%)	59 (100%)	24 (41%)
	Total duration of outbreak	82	01 November 2003 to 22 January 2004	86	50 (58%)	16 (19%)	86 (100%)	24 (28%)

**Table 3:** Relative risk of each source of contamination to be the source of infection for each wave of the outbreak.

Sources	Wave 1	Wave 2	Wave 3
Wastewater basin	50%	0.1%	96.98%
Cooling tower	50%	99.9%	2.91%
High-pressure water cleaner			0.1%
Car wash station			0.01%

## Data Availability

Data is contained within the article.
